# *motifbreakR* v2: expanded variant analysis including indels and integrated evidence from transcription factor binding databases

**DOI:** 10.1093/bioadv/vbae162

**Published:** 2024-10-23

**Authors:** Simon G Coetzee, Dennis J Hazelett

**Affiliations:** Department of Computational Biomedicine at Cedars-Sinai Medical Center, West Hollywood, CA 90069, United States; Department of Computational Biomedicine at Cedars-Sinai Medical Center, West Hollywood, CA 90069, United States

## Abstract

**Motivation:**

*motifbreakR* scans genetic variants against position weight matrices of transcription factors (TFs) to determine the potential for the disruption of binding at the site of the variant. It leverages the Bioconductor suite of software packages and annotations to query a diverse array of genomes and motif databases. Initially developed to interrogate the effect of single-nucleotide variants on TF binding sites, in *motifbreakR* v2, we have updated the functionality.

**Results:**

New features include the ability to query other types of complex genetic variants, such as short insertions and deletions. This capability allows modeling a more extensive array of variants that may have significant effects on TF binding. Additionally, predictions based on sequence preference alone can indicate many more potential binding events than observed. Adding information from DNA-binding sequencing datasets lends confidence to motif disruption prediction by demonstrating TF binding in cell lines and tissue types. Therefore, *motifbreakR can directly query* the ReMap2022 database for evidence that a TF matching the disrupted motif binds over the disrupting variant. Finally, in *motifbreakR*, in addition to the existing interface, we implemented an R/Shiny graphical user interface to simplify and enhance access to researchers with different skill sets.

**Availability and implementation:**

*motifbreakR* is implemented in R. Source code, documentation, and tutorials are available on Bioconductor at https://bioconductor.org/packages/release/bioc/html/motifbreakR.html and GitHub at https://github.com/Simon-Coetzee/motifBreakR.

## 1 Introduction

The consequences of genetic variants for transcription factor (TF) binding profoundly influence their impact on gene regulation. Predictions of these consequences therefore shape entire fields of study, from evolutionary genetics to human disease risk assessment. Tools and workflows have become increasingly sophisticated using machine learning and massively parallel reporter assays. However, it remains practical to maintain software libraries that can generate predictions based on position weight matrix (PWM) based matching analysis as a first-pass hypothesis generator or part of a larger bioinformatics workflow. *motifbreakR* remains relevant, having been used in many studies of human disease, from neurodegenerative disorders to research of coronavirus disease of 2019 (COVID-19). Additionally, it has been used in a variety of studies of model organisms, including yeast, mice, pigs, and blind cavefish (e.g. see D’Aurizio *et al.* 2022, Linder *et al.* 2022, Krishnan *et al.* 2022, Li *et al.* 2022, Mononen *et al.* 2024). It has even been used in paleoanthropology (Vespasiani *et al.* 2022) and evolutionary genomics (Kuderna *et al.* 2024).

*motifbreakR* allows for standardized analysis of any genome within the Bioconductor ecosystem, using any available PWM library and accepting various variant input formats, such as BED, VCF, lists of rsIDs, and custom variants in a flexible BED-derived format. *motifbreakR* v1 (Coetzee *et al.* 2015), did not support short insertion-deletion (indel) polymorphisms or multi-nucleotide polymorphisms. In *motifbreakR* v2, we have introduced support for these variant types, which together account for approximately 17% of all variants identified in whole-genome sequencing data from gnomAD and about 23% of common variants (global MAF > 0.01) annotated in dbSNP. Incorporating published ChIP-seq data further enhances the impact of *motifbreakR*’s predictions by providing evidence of TF binding. In this update, we document these enhancements along with other newly implemented features in *motifbreakR v2*, including a graphical web interface and new export formats designed for accessibility to non-specialist users.

## 2 Features

### 2.1 Insertion–deletion variants

We have implemented the import and analysis of indel variants to allow for querying a broader array of variations that alter TF binding in the genome. Variants are scored by scanning a motif across the reference and the alternate sequence; the returned score is the highest-scoring match (or, equivalently, the match with the lowest *P*-value) in the whole sequence. The effect size is the difference between the best match on the reference allele and the alternate allele.

**Figure 1.**
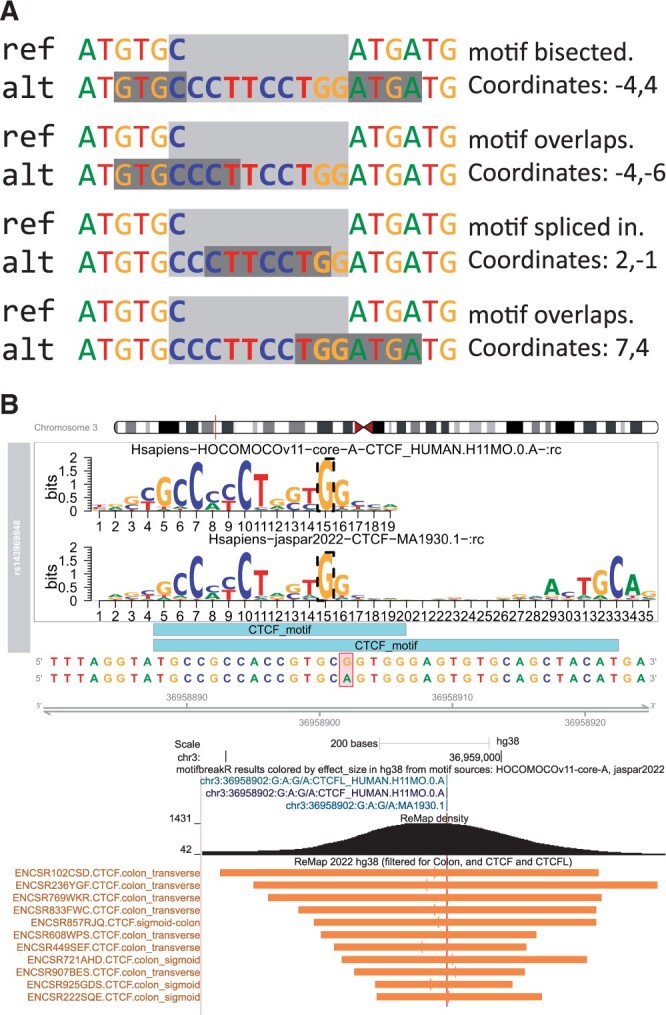
(A) Here, we represent four distinct ways that an indel can interrupt a motif and the coordinates that describe the position of the motif relative to the indel. A dark gray box highlights the consensus sequence of the motif, and the light grey box indicates the position of the indel. (B) A genome browser shot showing the export of motifbreakR results colored by the effect size of the variant. A darker color indicates a stronger effect. The remaining tracks illustrate native tracks available on the UCSC genome browser, “ReMap density” (the density of ReMap2022 results), and a representative sample of individual ChIP-Seq binding peaks for CTCF in colon samples from the same browser track. The motifbreakR results indicate the potential for a CTCF motif to be disrupted by the alternate allele of the variant, and ReMap2022 indicates evidence that CTCF can be found binding to the site of the variant.

The biggest challenge in implementation is defining a coherent coordinate system for specifying the position of the matching motif. In an example where an insertion is longer than the sequence of the queried PWM, the motif could be spliced and thus destroyed by the insertion. Alternatively, it creates the motif such that PWM overlaps the beginning, is entirely contained within, or overlaps the end of the insertion. In the instances where the motif is created, the position cannot be described by coordinates on the reference genome. *motifbreakR* treats insertions and deletions equally. An insertion in the reference allele is equivalent to a deletion in the alternate allele and is managed with the same coordinate system. *motifbreakR* defines the coordinates of a motif relative to the edges of the indel. The first number is the number of bases upstream (negative) or downstream (positive) of the start of the variant describing where the motif starts. The second number is the number of bases upstream or downstream of the end of the variant, indicating where the motif ends (Fig. 1A).

### 2.2 Querying of TF binding database

*motifbreakR* predicts differential TF binding based on sequence preference. However, integrating observed TF binding data can improve result prioritization. To achieve this, we have incorporated the ReMap2022 database of DNA-binding sequencing datasets, which provides *in vivo* evidence of TF binding at the variant sites identified by *motifbreakR*. Although this dataset does not cover every relevant cell type or developmental stage, it can provide binding evidence for 5% to 10% of results where *P *<* *1e−4. *motifbreakR* leverages this flexibility, allowing users to query ReMap2022 data for multiple species, including *Homo sapiens* (hg38, or hg19 as liftover), *Mus musculus* (mm10, or mm39 as liftover), *Drosophila melanogaster* (dm6), and *Arabidopsis thaliana* (TAIR10). Additionally, users can incorporate private or custom TF binding data by adapting the layout of ReMap2022 BED files, extending this functionality to any species supported by Bioconductor.

The new functions empower the user to build a local copy of the TF binding database and query a *motifbreakR* result. Once built, *motifbreakR* can rapidly annotate its results with ReMap sourced TF peaks corresponding to motif/TF relationships provided by the constituent public *MotifDb* (Shannon and Richards 2024) sources. The user may optionally query an expanded motif/TF relationship encompassing the entire potential TF family as implemented by *MotifDb* based on *TFClass* (Wingender *et al.* 2015, Shannon and Richards 2024). Though not comprehensive, including the experiment biotype (cell lines and tissue types) from ReMap may also be helpful for hypothesis generation. Figure 1B shows an example of a variant rs143969848 that breaks a CTCF motif centered on a ChIP-seq peak in multiple cell lines.

### 2.3 Graphical user interface

To make *motifbreakR* accessible to bench scientists, researchers with various skill sets, and others wishing to explore its capabilities on the web, we developed an R/Shiny-based graphical user interface (GUI) that facilitates all functions of the underlying *motifbreakR* package. In a workflow mirroring the code-based method, the user specifies individual rsIDs or “custom” variants and performs downstream tasks via pulldown menus and radio buttons. Like the R package version, users may upload VCFs or lists of single nucleotide variants (SNVs) in BED format. Any figures generated are downloadable/saved to the local environment. Finally, to promote reproducible science, analysis performed with the GUI will be output as code in R markdown format for publication.

### 2.4 Useful exports

*motifbreakR* now exports results in various tabular formats compatible with database programs, including Excel or SQL. In addition, *motifbreakR* can export a BED file of user-defined subsets of top matches for display in browsers such as UCSC genome browser (Nassar *et al.* 2023), WashU Epigenome browsers (Li *et al.* 2022), or IGV (Thorvaldsdóttir *et al.* 2012). Optionally, matches can be color-coded by motif quality (*P*-value-oriented) or disruptiveness (score-oriented).

## 3 Conclusion

We have updated *motifbreakR* to version 2 to include several new useful features, most notably indels, a new analysis pipeline to reference published ChIP-seq experiments that match *motifbreakR* predictions, and a GUI to promote accessibility and reproducibility.

## Data Availability

There are no primary data generated in this manuscript.
